# Continuous Hidden Markov Model Based Spectrum Sensing with Estimated SNR for Cognitive UAV Networks

**DOI:** 10.3390/s22072620

**Published:** 2022-03-29

**Authors:** Yuqing Feng, Wenjun Xu, Zhi Zhang, Fengyu Wang

**Affiliations:** 1 The State Key Laboratory of Networking and Switching Technology, Beijing University of Posts and Telecommunications, Beijing 100876, China; fyq@bupt.edu.cn (Y.F.); zhangzhi@bupt.edu.cn (Z.Z.); 2 The Key Lab of Universal Wireless Communications, Ministry of Education, Beijing University of Posts and Telecommunications, Beijing 100876, China; 3 School of Artificial Intelligence, Beijing University of Posts and Telecommunications, Beijing 100876, China; fengyu.wang@bupt.edu.cn

**Keywords:** cognitive UAV networks, clustered two-stage-fusion cooperative spectrum sensing, continuous hidden Markov model, SNR estimation

## Abstract

In this paper, to enhance the spectrum utilization in cognitive unmanned aerial vehicle networks (CUAVNs), we propose a cooperative spectrum sensing scheme based on a continuous hidden Markov model (CHMM) with a novel signal-to-noise ratio (SNR) estimation method. First, to exploit the Markov property in the spectrum state, we model the spectrum states and the corresponding fusion values as a hidden Markov model. A spectrum prediction is obtained by combining the parameters of CHMM and a preliminary sensing result (obtained from a clustered heterogeneous two-stage-fusion scheme), and this prediction can further guide the sensing detection procedure. Then, we analyze the detection performance of the proposed scheme by deriving its closed-formed expressions. Furthermore, considering imperfect SNR estimation in practical applications, we design a novel SNR estimation scheme which is inspired by the reconstruction of the signal on graphs to enhance the proposed CHMM-based sensing scheme with practical SNR estimation. Simulation results demonstrate the proposed CHMM-based cooperative spectrum sensing scheme outperforms the ones without CHMM, and the CHMM-based sensing scheme with the proposed SNR estimator can outperform the existing algorithm considerably.

## 1. Introduction

With the advantage of high flexibility and low deployment cost, unmanned aerial vehicles (UAVs) have been widely used in military communications, weather monitoring, emergency rescue [1] and some other UAV-assisted Internet of Things (IoT) applications [2]. The large-scale deployment of UAVs has exacerbated the shortage of spectrum resources. However, the existing spectrum allocation strategies cannot effectively use the scarce spectrum resources, which becomes the bottleneck for enhancing the communication performance of UAVs [3]. Cognitive radio (CR) is proposed to solve the problem, which improves the spectrum efficiency by perceiving spectrum holes and providing secondary UAVs with opportunities to reuse idle spectrum. Thus, CR can further guide the spectrum utilization in cognitive unmanned aerial vehicle networks (CUAVNs), including the resource allocation for low-latency communications [4], high-quality services with limited resources [5], maximum achievable throughput [6], and optimal power allocation. To enable CUAVNs, accurate spectrum sensing attaches great importance.

With the development of UAVs, spectrum sensing in unmanned aerial vehicle networks (UAVNs) has attracted attention from both academia and industry. The detection performance is enhanced by using multiple secondary UAVs in [7,8]. Authors in [3,9] consider the combination of UAVs and terrestrial communication equipment for air-ground-integrated spectrum sensing. The heterogeneity of spatial information is further taken into account by using the 3D [10], so as to improve the spectrum sensing detection performance. The above studies introduce information from multiple users and spatial dimensions to improve the spectrum utilization of CUAVNs. However, they do not consider the temporal information of the spectrum, which affects the detection performance of UAVNs. Exploring the temporal correlation of spectrum states by focusing on the Markov property is an effective and novel idea to enhance the detection probability [11].

Another concern is that knowledge of the signal-to-noise ratio (SNR) is required before spectrum sensing [12], e.g., energy detection and cyclostationary feature detection. Therein, it is assumed that SNR is perfectly known. However, UAVs need to move across a large area in lots of applications, and the links between two UAVs that are far apart frequently break and reestablish [13], which degrades the sensing performance and the spectrum utilization. Besides, large flying areas, unstable links, and dynamic network topologies lead to variable SNRs in CUAVNs, which makes the assumption of pre-known SNRs no longer applicable, bringing new challenges for sensing in UAVNs.

To overcome these problems, we propose a continuous hidden Markov model (CHMM) based sensing scheme with a novel space smoothing second- and fourth-order moments (SS-M2M4) SNR estimator. Note that the true states of the unauthorized spectrum are not observable, however, the sensing results originating from the unobservable spectrum states can be easily obtained. Therefore, a hidden Markov chain model fits well with the spectrum sensing procedure. To fully exploit the temporal information and further enhance the detection performance, we combine the spectrum sensing scheme with CHMM. To the best of our knowledge, our work is the first to consider the continuous hidden Markov model in non-centralized CUANs. Moreover, we further provide theoretical analysis of the detection probability and the false alarm probability, while previous works mainly focus on numerical simulations. Besides, for generalized estimation and higher accuracy, based on a general SNR estimation method of a second- and fourth- moments (M2M4) estimator [14], we propose a SS-M2M4 SNR estimator. The SNR smoothness of the neighboring CUAVs has been taken into account, enlightened by the smoothness of the graph signal [15]. Compared to the widely-used M2M4 estimator, our proposed SNR estimator achieves more accurate estimation. With the SNR provided by the proposed estimator, our CHMM-based spectrum sensing scheme can achieve a high detection probability. Our contributions are summarized as follows:

- We propose a spectrum sensing scheme based on a continuous hidden Markov model in CUAVNs to obtain better sensing performance.
- We derive the closed-form detection probability and false alarm probability expressions of the proposed CHMM-based spectrum sensing.
- Considering the fading similarity within the neighboring CUAVs in practical applications, we propose an SNR estimation scheme based on signal smoothness, which reduces the SNR estimation error effectively, and further enhances the performance of CHMM-based cooperative spectrum sensing.

The remainder of this paper is organized as follows. Section 2 discusses the related work. Section 3 introduces the system model. In Section 4, the CHMM based spectrum sensing scheme is proposed. In Section 5, the closed-form detection probability and false alarm probability expressions of the proposed method are derived. In Section 6, we design a novel SNR estimator to enhance the proposed CHMM-based spectrum sensing scheme with a more accurate SNR. The simulation results are presented to evaluate the proposed algorithms in Section 7. Section 8 concludes the work and discusses the possible future work.

## 2. Related Work

### 2.1. Hidden Markov Model (HMM) Based Spectrum Sensing

Hidden Markov model (HMM) based spectrum sensing means using the HMM to model the spectrum sensing procedure. As the HMM has a hidden layer and an observable layer, it fits well with the sensing procedure whose spectrum states are unknown but the receiver can be obtained. Exploring the Markov property of the spectrum states is an effective way to enhance the sensing performance [11]. Compared with deep learning based spectrum sensing [16,17], CHMM-based spectrum sensing has a stronger interpretability and smaller delay, and the initial probability distribution can be used to calculate the initial spectrum utilization. In addition, the obtained parameters can also be used for digital twinning of the communication system. It has been validated that the spectrum state can be modeled as a Markov chain by analyzing real-world measurements [18]. A hidden Markov model based scheme [19] is proposed to predict the arrival of the primary user (PU). Authors in [20] evaluate the reliability of HMM based cooperative spectrum sensing in cognitive radio networks, in the presence of random malfunctioning of secondary user nodes participating in the process. Occupancy prediction schemes based on a discrete hidden Markov model (DHMM) and a continuous hidden Markov model are investigated in [21,22,23,24], respectively. Authors in [21,22] adopt DHMM to model the spectrum sensing procedure, which do not make full use of the information obtained by the receiver. Authors in [23,24] use CHMM to model the sensing procedure of CUAVNs. However, they do not consider the dynamicity of UAVs, which is an important characteristic of the UAV networks [25]. Besides, centralized spectrum sensing methods in [23,24] do not work well in CUAVNs since the global fusion center is usually unreachable to secondary CUAVs. Thus, in this paper, we propose a CHMM-based spectrum sensing method to enhance the spectrum utilization in CUANs.

### 2.2. SNR Estimation

SNR estimation refers to the calculation of SNR by using signal information [14]. Various algorithms require SNR estimation for optimal performance if the SNR is not constant, such as linear diversity combining techniques and Viterbi algorithms with soft-decision [14]. Note that the knowledge of SNR is also required for typical commonly-used spectrum sensing [12]. However, in practical UAV applications, it is difficult to obtain accurate SNR. Conventionally, SNR estimators require knowledge of the signal or the channel, such as the maximum likelihood (ML) SNR estimator [26] and particle swarm optimization (PSO) SNR estimator, based on parameters of hardwares or channels [27]. In addition, there exist estimators designed for specific signals or specific feature spectrum sensing methods, such as the SNR estimator [28] for M-ary amplitude phase shift keying (M-APSK) modulated signals, the SNR estimator for signal with Polar code [29], and the estimator for eigenvalue-based spectrum detectors [30]. Lacking the prior knowledge of the signal/channel and design for specific signals/sensing methods make it hard to generally adopt the above estimators to various CUAVNs. Besides, some scholars have paid attention to deep learning based SNR estimation methods, such as the convolutional neural networks (CNN)—long short term memory (LSTM) based SNR estimators [31] and the CNN-based SNR estimators designed for UAVNs [32]. However, in the spectrum sensing of CUAVNs, the deep learning based methods are too complicated and take more time, leading to the sensing term being missed. Taking all the above into consideration, we design a generalized and low-complexity SNR estimator named SS-M2M4. Based on the proposed SNR estimator, the CHMM-based spectrum sensing method can further enhance detection performance.

## 3. System Model

**Figure 1:**
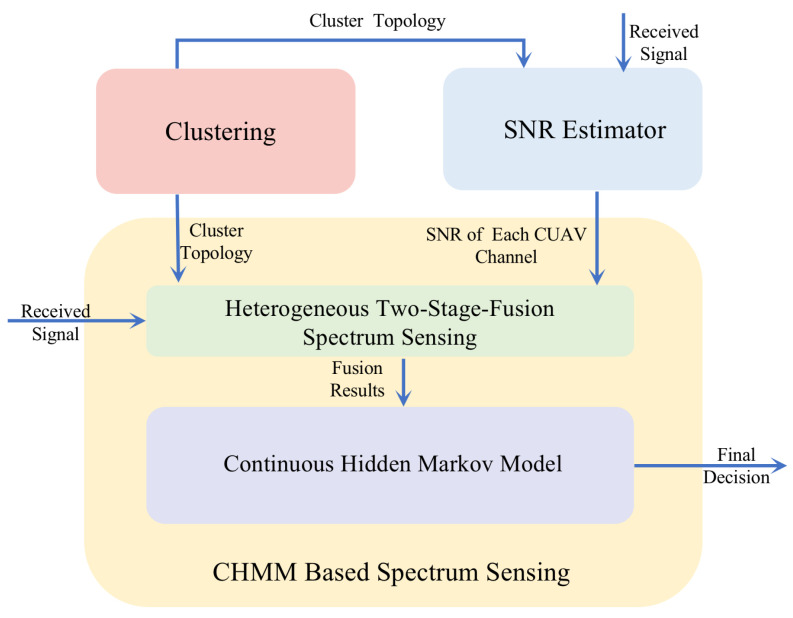
The unified framework of the CHMM-based spectrum sensing scheme with the SS-M2M4 estimator.

In order to detect whether the authorized spectrum of PU is occupied, a spectrum sensing method is adopted by multiple UAVs [3,33]. In this paper, we propose a CHMM-based spectrum sensing method with consideration of the imperfect SNR estimation to enhance the detection probability. The overview of this unified scheme is shown in Figure 1. First, to obtain the single-time spectrum sensing fusion result, we adopt the max-min distance clustering algorithm [34] and heterogeneous two-stage-fusion spectrum sensing scheme [35], which are described in Section 3.1 and Section 3.2, respectively. Then, to fully take advantage of the temporal correlation of the spectrum states, we propose a CHMM-based spectrum sensing method, which will be introduced in Section 4. Considering the imperfect SNR estimation in practical applications, we propose a novel SNR estimator shown in Section 6 to offer the sensing scheme more desirable SNRs. Finally, combining the CHMM-based spectrum sensing and the proposed SNR estimator, we propose the unified CHMM-based spectrum sensing scheme with advanced SNR estimator.

### 3.1. Clustering Method

Considering the fusion delay brought by distributed cooperative spectrum sensing (DCSS), and to further provide the proposed SS-M2M4 SNR estimator with graph topology, a max-min distance clustering algorithm [34] is adopted. It divides CUAVs with similar position and mobility into the same cluster, and selects the one with the highest trust value in each cluster as the cluster head. Thus, we can adopt intra-cluster centralized cooperative spectrum sensing. Due to the similar locations, the UAVs in the same cluster have similar SNRs, which facilitates the SNR estimation. The cluster heads are less than the number of the CUAVs, so the fusion delay will be reduced and, therefore, we adopt inter-cluster distributed cooperative spectrum sensing.

### 3.2. Heterogeneous Two-Stage-Fusion Spectrum Sensing

**Figure 2:**
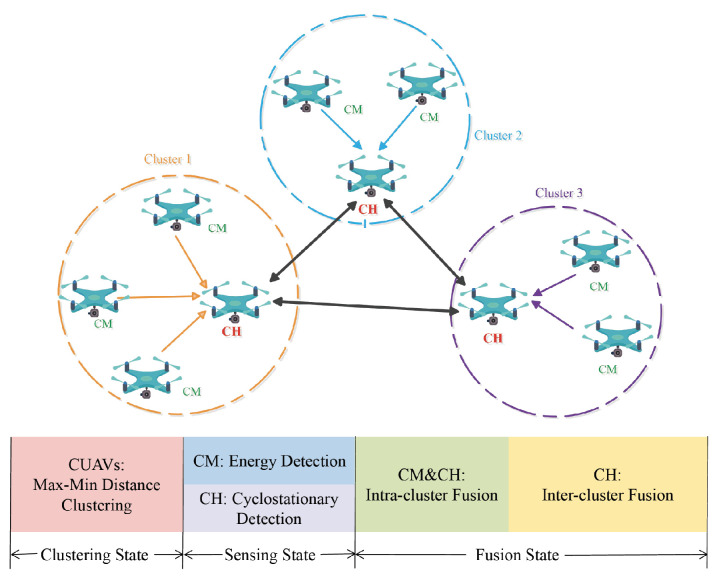
Heterogeneous Two-Stage-Fusion.

To further improve the sensing performance in CUAVNs, a heterogeneous cooperative spectrum sensing scheme is employed with the clustering outcome, as shown in Figure 2. A symbol table of the notations is shown in Table 1.

#### 3.2.1. Heterogeneous Spectrum Sensing Based on Clustering

To be specific, in the sensing state, a heterogeneous cooperative spectrum sensing scheme [36] is used according to the clustering result. Here, the "heterogeneous” means two different detection schemes: energy detection and cyclostationary detection [36]. Cluster heads adopt cyclostationary detection, while other secondary UAVs in the same cluster adopt energy detection. Cyclostationary feature detection is adopted to the cluster head, since it has great detection accuracy and can maintain good performance even in environments with low SNR. The cluster members (CMs) adopt energy detection, since it is simple to implement and does not require prior knowledge of the channels. Similarly to [36], we assume that the transmitted signal of the primary user is a sinusoidal signal, in which the n-th sample is expressed as

(1) $$\begin{array}{c} s\left(n\right)=ae^{j2\pi f_{c}n+\varphi }. \end{array}$$

where *a* is the amplitude, $f_{c}$ is the carrier frequency, and φ is the carrier phase offset. The received signal of the cluster heads is written as

(2) $$\begin{array}{c} y_{C}\left(n\right)=\left\{\begin{array}{cc} \omega (n) & H_{0}\\ s(n)+\omega (n) & H_{1} \end{array}\right., \end{array}$$

where ω(n) denotes the additive Gaussian noise with zero mean and unit variance. $H_{0}$ indicates that the spectrum is absent, while $H_{1}$ indicates that the spectrum is occupied. The test statistic for the first-order cyclostationary detection [36] is expressed as

(3) $$\begin{array}{c} T_{C}=\left|\frac{1}{M}\sum_{n=1}^{M}y_{C(n)}e^{-j2\pi \alpha n}\right|, \end{array}$$

where α is the cyclic frequency, *M* denotes the number of sampling point. The received signal $y_{E}\left(n\right)$ of the CUAV cluster member is modeled as the same as $y_{C}\left(n\right)$

(4) $$\begin{array}{c} y_{E}\left(n\right)=\left\{\begin{array}{cc} \omega (n), & H_{0}\\ s(n)+\omega (n), & H_{1} \end{array}\right.. \end{array}$$

The test statistic for the energy detector [36] is expressed as

(5) $$\begin{array}{c} T_{E}=\frac{1}{M}\sum_{n=1}^{M}{\left|y_{E}\left(n\right)\right|}^{2}, \end{array}$$

where *M* denotes the number of sampling points. Let $T_{ic}=\left[T_{C_{i}},T_{E_{i1}},T_{E_{i2}},\ldots ,T_{E_{iK_{E}}}\right]$ denotes the vector of the statistics from the *i*th cluster, $T_{C_{i}}$ is the cyclostationary detection statistics of the *i*th cluster head, and its distribution [36] can be expressed as

(6) $$\begin{array}{c} T_{C_{i}}∼\left\{\begin{array}{cc} \mathrm{FN}(\sqrt{\frac{2}{\pi M}},\frac{\pi -2}{\pi M}), & H_{0}\\ \mathrm{FN}(\zeta_{i},2\gamma_{C_{i}}+\frac{1}{M}-\zeta_{i}^{2}), & H_{1} \end{array}\right., \end{array}$$

where $\zeta_{i}=\sqrt{\frac{2}{\pi M}}e^{-M\gamma_{C_{i}}}+\sqrt{2\gamma_{C_{i}}}\left(1-2Q\left(\sqrt{2M\gamma_{C_{i}}}\right)\right)$, $\gamma_{C_{i}}$ denotes the SNR of the *i*th cluster head, and FN is the folded normal distribution [37]. $T_{E_{ij}}$ is the energy detection statistics of the *j*th the node in the *i*th cluster. When *M* is large enough, $T_{E_{ij}}$ approximatively follows normal distribution according to the central limit theorem [38], but slightly different to [38], our $T_{E_{ij}}$ is 1/*M* times the energy detection statistics in [38], thus the our SNR is *M* times the SNR in [38], and we assume the energy of the received signal is 1. Thus, the distribution of $T_{E_{ij}}$ can be expressed as

(7) $$\begin{array}{c} T_{E_{ij}}∼\left\{\begin{array}{cc} N({\gamma_{E_{ij}}}^{-1},\frac{2{\gamma_{E_{ij}}}^{-2}}{M}), & H_{0}\\ N(1+\gamma_{E_{ij}}^{-1},\frac{2(\gamma_{E_{ij}}^{-2}+2{\gamma_{E_{ij}}}^{-1})}{M}), & H_{1} \end{array}\right., \end{array}$$

where $\gamma_{E_{ij}}$ is the SNR of the *j*th node in the *i*th cluster.

#### 3.2.2. Two-Stage-Fusion

In the fusion duration, we adopt a two-stage-fusion scheme [34], which includes the intra-cluster fusion stage and the inter-cluster fusion stage. The CUAV cluster members adopt energy detection, and we apply high-accuracy centralized soft fusion in the intra-cluster since the performance of energy detection is somewhat not precise. In the intra-cluster fusion stage, according to the assumption that the observations are independent, we can attain the likelihood ratio test (LRT) [36] of the *i*th cluster

(8) $$\begin{array}{cc} T_{i} & =\prod_{j=1}^{K}\frac{P[T_{ij}|H_{1}]}{P[T_{ij}|H_{0}]}\\ \; & =\prod_{j=1}^{K_{E}}\frac{P[T_{E_{ij}}|H_{1}]}{P[T_{E_{ij}}|H_{0}]}\times \frac{P[T_{Ci}|H_{1}]}{P[T_{Ci}|H_{0}]}, \end{array}$$

there are *K* CUAVs, $K_{E}$ CMs in the *i*th cluster, where $K_{E}=K-1$. $T_{ij}$ denotes cyclostationary detection statistics or energy detection statistics, $P[T_{ij}|H_{1}]$ and $P[T_{ij}|H_{0}]$ represent the probability density under hypotheses $H_{1}$ and $H_{0}$, respectively. According to the $T_{E_{ij}}$, $T_{C_{i}}$, the LRT in Equation (8) can be simplified [36] as

(9) $$\begin{array}{c} T_{i}=\sum_{j=1}^{K_{E}}\omega_{ij}T_{E_{ij}}+\rho_{i}T_{Ci}. \end{array}$$

where $\omega_{ij}=\frac{\gamma_{E_{ij}}}{2(1+\gamma_{E_{ij}})}$, and $\rho_{i}=\sqrt{2M^{2}\gamma_{C_{i}}}$.

As for the inter-cluster fusion stage, considering the good sensing performance of cyclostationary detection and the large distance between the cluster heads, distributed consensus-based fusion [38] is performed. Each cluster head communicates with its neighbouring cluster heads to exchange information, and the exchange process is iteratively done. The initial information (which is the intra-cluster fusion result) of the *i*th cluster head is denoted as $T_{i}\left(0\right)$. Then, according to the network topology, these cluster heads repeatedly iterate until $T_{i}\left(k\right)$ covers a common value. The consensus-based scheme [38] is

(10) $$\begin{array}{c} T_{i}\left(k+1\right)=WT_{i}\left(k\right). \end{array}$$

where $W=I-\alpha \Delta^{-1}L$, and *L* is the Laplacian matrix of the cluster heads topology. α is the step size, and it satisfies $0<\alpha <d^{-1}$, *d* is maximum node degree of the graph. $\Delta =diag\{\delta_{h_{1}},\delta_{h_{2}},\ldots ,\delta_{h_{n}}\}$, where $\delta_{hi}$ [38] is the weight according to the channel condition of the *i*th cluster head, and it satisfies $\delta_{h_{i}}\geq 1$. The cluster heads communicate with their own neighbors, then a final consensus [38] is reached as

(11) $$\begin{array}{c} T^{*}=\lim_{k\to \infty }T\left(k\right)=\frac{\sum_{i=1}^{n}\delta_{h_{i}}T_{i}\left(0\right)}{\sum_{h_{i=1}}^{n}\delta_{h_{i}}}. \end{array}$$

## 4. Continuous Hidden Markov Model Based Spectrum Sensing

**Figure 3:**
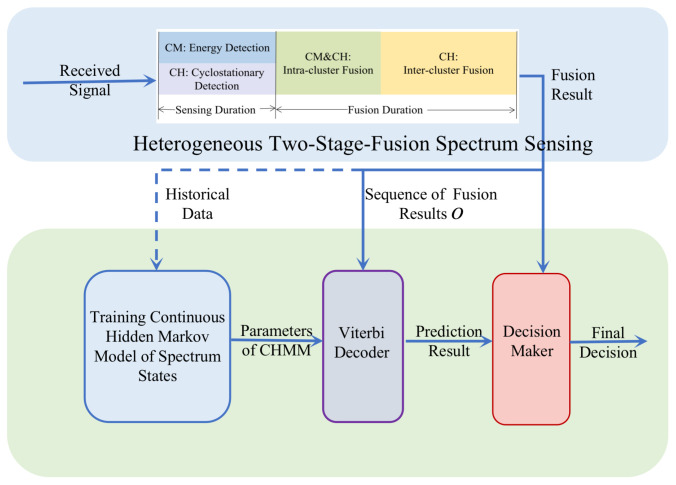
CHMM-based Spectrum Sensing.

To fully take advantage of the temporal correlation of the spectrum states, we propose a CHMM-based spectrum sensing scheme as shown in Figure 3. Firstly, hidden HMM and CHMM are introduced, and the suitability regarding the spectrum states as a continuous hidden Markov model are analyzed. Then, the model is trained with the forward-back algorithm and Baum–Welch algorithm. The prediction obtained according to CHMM model is used to assist the sensing.

### 4.1. Hidden Markov Model

The hidden Markov model is a double stochastic process with a hidden layer and an observable layer. The hidden process is an unobservable Markov chain, which can be obtained through the observed states.

The true states of the spectrum are not observable but the sensing results can be easily obtained. Therefore, the hidden Markov chain model fits well with the PU spectrum state. For HMM, there are three basic problems that need to be solved, i.e., the evaluation problem that computes the probability of the observed fusion result sequence, the learning problem that adjusts the model parameters to maximize the probability of the observed sequence, and the predication problem that calculates the most likely hidden spectrum state sequence according to the observation sequence and model parameters.

In order to avoid the distortion caused by the discretization of continuous variables in the cluster heads, we consider the continuous HMM, which replaces the discrete observation states with continuous characteristics. With more specific spectrum information, we can obtain better detection performance.

### 4.2. Continuous Hidden Markov Model of Spectrum States

**Figure 4:**
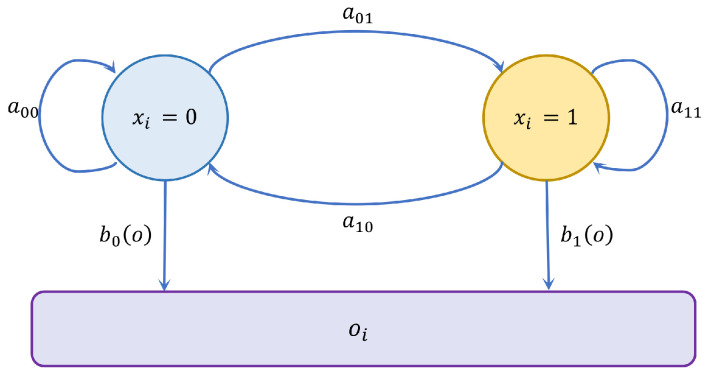
Continuous hidden Markov model.

The PU spectrum state at time instant *t* is given by $x_{t}$, and it can be 0 or 1, where 0 represents spectrum absence, 1 denotes spectrum occupancy. The sequence of the PU states $X=\left(x_{1},x_{2},\ldots ,x_{t}\right)$ can be seen as the hidden Markov chain. $o_{t}$ is the fusion value of the heterogeneous two-fusion-stage spectrum sensing at time instant *t*, and $O=\left(o_{1},o_{2},\ldots ,o_{t}\right)$ is the observable layer. The hidden Markov chain and the observable layer constitute a continuous hidden Markov model, which can be formulated as λ=(π,A,μ,Σ,C) [39], where π represents the initial probability vector of the hidden spectrum state, *A* is the transition matrix of the two states. The continuous hidden Markov model can be represented in Figure 4. μ,Σ,C are the parameters of the observation probability distribution. The Gaussian mixture model (GMM) is used to model the probability, as the Gaussian process has good adaptability in dealing with complex regression problems and classification. Thus, the observation probability in state *i* according to the GMM can be written as

(12) $$\begin{array}{c} b_{i}\left(o\right)=\sum_{m=1}^{M}C_{im}N\left(o,\mu_{im},\Sigma_{im}\right),i=0,1, \end{array}$$

which is composed of *M* Gaussian mixtures. *i* denotes the spectrum state. $C_{im}$ is the proportion of the *m*th mixture coefficient in state *i*, *o* denotes the fusion result calculated by the clustered heterogeneous two-fusion-stage scheme, $\mu_{im}$ and $\Sigma_{im}$ represent the mean and the covariance of *m*th mixture in state *i*, respectively.

To employ the continuous hidden Markov model to cognitive UAV networks, the forward-backward algorithm and Baum–Welch algorithm are utilized to solve the evaluation problem and the training problem, respectively. As for the prediction problem, the Viterbi algorithm is utilized.

### 4.3. Evaluation Process and Learning Process of Continuous Hidden Markov Model

For the evaluation problem, the forward-back algorithm is used to calculate the probability of the observed fusion value sequence, and it can be divided into two parts: the forward algorithm and backward algorithm. For a given λ and the spectrum state at time *t*, the forward quantity $\alpha_{t}\left(i\right)$ is the joint probability of sequence *O* from the initial time to time *t*, and the state in $S_{i}$ at time *t*, $\beta_{t}\left(i\right)$ is the joint probability of sequence from time t+1 to the final time and the state in $S_{i}$ at time *t*. The forward and backward quantities [39] are defined as

(13) $$\begin{array}{c} \alpha_{t}\left(i\right)=P\left(o_{1},o_{2},\ldots ,o_{t},q_{t}=S_{i}|\lambda \right), \end{array}$$

(14) $$\begin{array}{c} \beta_{t}\left(i\right)=P\left(o_{t+1},o_{t+2},\ldots ,o_{T}|q_{t}=S_{i},\lambda \right), \end{array}$$

with the initializations

(15) $$\begin{array}{c} \alpha_{1}\left(i\right)=\pi_{i}b_{i}\left(o_{1}\right),i=0,1, \end{array}$$

(16) $$\begin{array}{c} \beta_{T}\left(i\right)=1,i=0,1. \end{array}$$

The forward and backward quantities can be calculated by

(17) $$\begin{array}{c} \alpha_{t}\left(j\right)=\left[\sum_{i=1}^{2}\alpha_{t}\left(i\right)a_{i,j}\right]b_{j}\left(o_{t}\right),t=1,2,\ldots ,T,j=0,1, \end{array}$$

(18) $$\begin{array}{c} \beta_{t}\left(i\right)=\sum_{j=1}^{2}a_{i,j}b_{j}\left(o_{t+1}\right)\beta_{t+1}\left(j\right),t=T-1,T-2,\ldots ,1,i=0,1. \end{array}$$

Then, combining the forward and backward algorithm, we can get the forward-backward algorithm, then the probability of the observed fusion result sequence *O* with the given model parameters λ is obtained [39] as

(19) $$\begin{array}{c} P\left(O|\lambda \right)=\sum_{i=1}^{2}\alpha_{t}\left(i\right)\beta_{t}\left(i\right). \end{array}$$

To solve the learning problem, the Baum–Welch algorithm is adopted, which is one of the expectation–maximization algorithms and uses the forward-backward algorithm in each expectation process. Before using this algorithm, we need to define three parameters: $\gamma_{t}\left(i\right)$, $\xi_{t}\left(i,j\right)$ and $\gamma_{t}\left(j,m\right)$. $\gamma_{t}\left(i\right)$ denotes the probability of the *i*th spectrum state at time *t*, $\xi_{t}\left(i,j\right)$ denotes the probability that at time *t* the spectrum state is $S_{i}$ and at time t+1 the spectrum state is $S_{j}$, $\gamma_{t}\left(j,m\right)$ denotes the probability of the *m*th Gaussian mixture of state $S_{j}$ at time *t*. $\gamma_{t}\left(i\right)$, $\xi_{t}\left(i,j\right)$ and $\gamma_{t}\left(j,m\right)$ can be calculated [39] as follows:

(20) $$\begin{array}{c} \gamma_{t}\left(i\right)=\frac{\alpha_{t}\left(i\right)\beta_{t}\left(i\right)}{\sum_{i=1}^{2}\alpha_{t}\left(i\right)\beta_{t}\left(i\right)}, \end{array}$$

(21) $$\begin{array}{c} \xi_{t}\left(i,j\right)=\frac{\alpha_{t}\left(i\right)a_{ij}b_{j}\left(o_{t+1}\right)\beta_{t+1}\left(j\right)}{\sum_{i=1}^{2}\sum_{j=1}^{2}\alpha_{t}\left(i\right)a_{ij}b_{j}\left(o_{t+1}\right)\beta_{t+1}\left(j\right)}, \end{array}$$

(22) $$\begin{array}{c} \gamma_{t}\left(j,m\right)=\frac{\alpha_{t}\left(j\right)\beta_{t}\left(j\right)}{\sum_{i=1}^{2}\alpha_{t}\left(i\right)\beta_{t}\left(i\right)}\cdot \frac{c_{j,m}N\left(o_{t},\mu_{j,m},\Sigma_{j,m}\right)}{\sum_{n=1}^{2}N\left(o_{t},\mu_{j,m},\Sigma_{j,m}\right)}. \end{array}$$

When enough training data are provided, that is, the sequences of fusion results obtained from the heterogeneous two-stage-fusion sensing scheme and corresponding spectrum states, the Baum–Welch algorithm offers a way to train the model, outputting good CHMM parameters. In specific, initial model parameters are first selected according to the spectrum condition. Second, $\alpha_{t}\left(i\right)$, $\beta_{t+1}\left(j\right)$, $\gamma_{t}\left(i\right)$, $\xi_{t}\left(i,j\right)$ and $\gamma_{t}\left(j,m\right)$ are calculated. Third, the parameters are updated according to Appendix A. The forward-backward procedure and the updating procedure are repeated until the probability of the observation sequence P(O|λ) satisfies the convergence condition or the increments of parameters are less than threshold. Finally, we can obtain the trained model parameters λ=(π,A,μ,Σ,C).

### 4.4. Predication of Spectrum State with CHMM

In this section, we adopt the Viterbi algorithm to solve the predication problem. With the learned model parameters and the observed sequence of fusion results, we can calculate the joint probability of the observed sequence. The real spectrum state sequence is calculated [39] as

(23) $$\begin{array}{c} P(O,X|\lambda )=P(O|X,\lambda )P(X,\lambda )\\ =\pi_{x_{1}}b_{x_{1}}\left(o_{1}\right)a_{x_{1}x_{2}}b_{x_{2}}\left(o_{2}\right)a_{x_{T-1}x_{T}}b_{x_{T}}\left(o_{T}\right), \end{array}$$

where $x_{t}$ denotes the spectrum state at time *t*, $a_{x_{t}x_{t+1}}$ represents the transition probability from state $x_{t}$ to $x_{t+1}$, $b_{x_{t}}\left(o_{t}\right)$ denotes the observation probability of $o_{t}$, when the real spectrum state is $x_{t}$. Then, select the sequence with the maximum probability as the prediction sequence

(24) $$\begin{array}{c} X=\underset{allX}{\mathrm{argmax}}P\left(O,X|\lambda \right). \end{array}$$

Next, we can get the prediction $x_{T}$ and the prediction sequence *X*, and compare the predication result X with the real state sequence to get the prediction accuracy $P_{r}$. When the prediction is “busy”, $P_{r}$ can denote the probability that the spectrum is really occupied at time *T*, $1-P_{r}$ can denote the probability that the spectrum is really absent at time *T*. Similarly, when the prediction is “idle”, the probability of occupancy is $1-P_{r}$, and the probability of absence is $P_{r}$.

Combining the prediction accuracy of cluster heads with the fusion result of the detectors, we can get a new false alarm probability and detection probability. There are mainly two kinds of predictions: busy and idle. When the prediction is “busy”, we multiply the fusion result *T* by ϵ(ϵ>1). Similarly, when the prediction result is “idle”, we multiply the fusion value by η(η<1). Then we obtain the final fusion statistic adjusted by the prediction. After that, we compare the final fusion statistic with the threshold. When the fusion statistic is larger than the threshold, the decision is “busy” and vice versa. Thus, the final detection probability $P_{D}$ can be calculated as

(25) $$\begin{array}{c} P_{D}=P_{r}\cdot P_{d}\left(\epsilon T\right)+\left(1-P_{r}\right)\cdot P_{d}\left(\eta T\right). \end{array}$$

## 5. Analysis of Detection Performance for CHMM-Based Spectrum Sensing

Under $H_{1}$ means the spectrum is occupied. When the sampling points are large enough, $T_{E_{ij}}$ approximately follows normal distribution, as does $T_{C_{j}}$ (which will be explained in Section 7.1). Thus, when the spectrum is occupied, the fusion result can be calculated as

(26) $$\begin{array}{cc} T & =\sum_{i=1}^{n}\delta_{h_{i}}\left(\sum_{j=1}^{K_{iE}}\omega_{ij}T_{E_{ij}}+\rho_{i}T_{Ci}\right)\\ \; & =\sum_{i=1}^{n}\sum_{j=1}^{K_{iE}}\delta_{h_{i}}\omega_{ij}T_{E_{ij}}+\sum_{i=1}^{n}\delta_{h_{i}}\rho_{i}T_{Ci}, \end{array}$$

where *n* is the number of clusters, $\delta_{hi}$ is the weight according to the channel condition of the *i*th cluster head, $K_{iE}$ is the number of cluster members (CMs) in the *i*th cluster. According to [36], the weight can be simplified as $\omega_{ij}=\gamma_{E_{ij}}/2(1+\gamma_{E_{ij}})$, and $\rho_{i}=\sqrt{2M^{2}\gamma_{C_{i}}}$. The fusion result *T* is an approximately normally distributed random variable with mean $\mu_{T}$ and variance ${\sigma_{T}}^{2}$, the mean and the variance are expressed as

(27) $$\begin{array}{cc} \mu_{T} & =\sum_{i=1}^{n}\sum_{j=1}^{K_{iE}}\delta_{h_{i}}\omega_{ij}\left(1+{\gamma_{E_{ij}}}^{-1}\right)+\sum_{i=1}^{n}\delta_{h_{i}}\rho_{i}\sqrt{2\gamma_{C_{i}}}\\ \; & =\sum_{i=1}^{n}\sum_{j=1}^{K_{iE}}\frac{\delta_{h_{i}}}{2}+\sum_{i=1}^{n}2M\delta_{h_{i}}\gamma_{Ci}, \end{array}$$

and

(28) $$\begin{array}{c} {\sigma_{T}}^{2}=\sum_{i=1}^{n}\sum_{j=1}^{K_{iE}}\frac{{\delta_{h_{i}}}^{2}\left(1+2\gamma_{E_{ij}}\right)}{2M{\left(1+\gamma_{E_{ij}}\right)}^{2}}+\sum_{i=1}^{n}2M{\delta_{h_{i}}}^{2}{\gamma_{Ci}}^{2}. \end{array}$$

Then we can obtain the final detection probability $P_{D}$ of the CUAVNs,

(29) $$\begin{array}{c} P_{D}=P_{r}\cdot Q\left(\frac{\frac{\lambda_{T}}{\epsilon }-\mu_{T}}{\sigma_{T}}\right)+\left(1-P_{r}\right)\cdot Q\left(\frac{\frac{\lambda_{T}}{\eta }-\mu_{T}}{\sigma_{T}}\right), \end{array}$$

where $\lambda_{T}$ is the threshold.

Under $H_{0}$, when *M* is large enough, $T_{E_{ij}}$ approximately follows normal distribution, and the probability density $T_{C_{ij}}$ can be approximately represented as

(30) $$\begin{array}{c} f\left(T_{C,i}\right)=\frac{2}{\sqrt{2\pi }\sigma_{T_{C,i}}}\exp\left\{-\frac{{T_{C,i}}^{2}}{2{\sigma_{T_{C,i}}}^{2}}\right\}. \end{array}$$

The probability density of the fusion result *T* can be represented as

(31) $$\begin{array}{cc} f(T) & =\frac{n}{\sqrt{2\pi \sigma^{2}}}\exp\left\{-\frac{{\left(T-\gamma \right)}^{2}}{2\sigma^{2}}\right\}\times erfc\left(\frac{\sigma_{C}\left(T-\gamma \right)}{\sqrt{2{\sigma_{E}}^{2}\sigma^{2}}}\right), \end{array}$$

where $\gamma =\sum_{i=1}^{n}\sum_{j=1}^{K_{E}}\delta_{h_{i}}/\left\{2\left(1+\gamma_{E_{ij}}\right)\right\}$, $\sigma^{2}={\sigma_{E}}^{2}+{\sigma_{C}}^{2}$, ${\sigma_{C}}^{2}=\sum_{i=1}^{n}2M{\delta_{h_{i}}}^{2}\gamma_{C_{i}}$, ${\sigma_{E}}^{2}=\sum_{i=1}^{n}\sum_{j=1}^{K_{E}}{\delta_{h_{i}}}^{2}/\left\{2M{\left(1+\gamma_{E_{ij}}\right)}^{2}\right\}$. The proof of f(T) is shown in Appendix B.

The false alarm probability can be calculated as follows, where $F\left(T\right)=\int_{0}^{T}f\left(x\right)dx$,

(32) $$\begin{array}{cc} P_{F} & =P_{r}\cdot \left(1-F\left(\eta T\right)\right)+\left(1-P_{r}\right)\cdot \left(1-F\left(\epsilon T\right)\right)\\ \; & =1-P_{r}F\left(\eta T\right)-\left(1-P_{r}\right)F\left(\epsilon T\right). \end{array}$$

## 6. CHMM-Based Spectrum Sensing with Practical SNR Estimation

The above work is based on the assumption that the SNR is perfectly known, i.e., we use a perfectly-known SNR when calculating the fusion weight, detection probability and false alarm probability. In this section, we further consider the scenario that the SNR is not perfectly known, and design a novel SNR estimation algorithm: space smoothing-based M2M4 (SS-M2M4), which modifies M2M4 with spatial smoothness, a technique that is used in the field of signal reconstruction in graphs. This algorithm can provide a more accurate SNR for the proposed CHMM-based spectrum sensing method.

### 6.1. Typical SNR Estimator

M2M4 is one of the most widely used blind estimators [14]. M2 and M4 represent the second and the fourth moment of $y_{n}$, respectively, where $y_{n}$ refers to the samples of the received signal. M2 and M4 can be calculated as follows [14]

(33) $$\begin{array}{cc} M_{2} & =E\{y_{n}y_{n}^{*}\}, \end{array}$$

(34) $$\begin{array}{cc} M_{4} & =E\{{\left(y_{n}y_{n}^{*}\right)}^{2}\}. \end{array}$$

Then with second-order moments and fourth-order moments, we can estimate SNR as follows,

(35) $$\begin{array}{c} \hat{p}=\frac{\sqrt{2M_{2}^{2}-M_{4}}}{M_{2}-\sqrt{2M_{2}^{2}-M_{4}}}. \end{array}$$

In practice, the second and fourth moments are usually calculated by their own time averages:

(36) $$\begin{array}{c} M_{2}=\frac{1}{M}\sum_{n=0}^{M}{y_{n}}^{2}, \end{array}$$

(37) $$\begin{array}{c} M_{4}=\frac{1}{M}\sum_{n=0}^{M}{y_{n}}^{4}. \end{array}$$

### 6.2. SS-M2M4 SNR Estimation Algorithm

In CUAVNs, the CUAVs within the same cluster are close to each other, and thus, their large-scale fading and shadow fading are generally similar [40]. Therefore, their SNRs are correlated. However, the M2M4 algorithm estimates these SNRs individually, ignoring the spatial correlation among the SNRs of those neighbouring users. CUAVNs have their own topology, and the SNR estimation problem can be naturally modeled as graph signal processing problems. Therefore, we propose a novel SNR estimation algorithm as shown in Algorithm 1, which considers the smoothness between neighbors [15]. Firstly, the M2M4 algorithm is applied to calculate the SNR of each CUAV, and the estimation result can be represented as $p_{0}=\left\{p_{01},p_{02},\ldots ,p_{0N}\right\}$. After that, to ensure that the secondary CUAVs that are close to each other are in the same cluster, the max–min distance clustering algorithm described in Section 3.1 is adopted. Based on the clustering result, we consider each cluster as a new graph, and then correct the original SNR to get the final estimated SNR $\hat{p}=\left\{{\hat{p}}_{1},{\hat{p}}_{2},\ldots ,{\hat{p}}_{N}\right\}$ by Equation (42).

The problem of estimating $\hat{p}$ from the original SNR $p_{0}$ can be modeled as the following optimization problem,

(38) $$\begin{array}{c} \min_{\hat{p}}\;\frac{1}{2}{\|p_{0}-\hat{p}\|}_{2}^{2}+\frac{\rho }{2}{\|H\hat{p}\|}^{2}, \end{array}$$

where H is a high-pass graph filter, and ρ is the regularization parameter. The first term penalizes the error of the estimated graph signal, the second term encourages the smoothness of the estimated SNR. Similar to [15], we set $H=L^{1/2}$, where L is the Laplacian matrix, so that ${\|H\hat{p}\|}_{2}^{2}={\hat{p}}^{T}L\hat{p}$. The smoothness of the estimated graph signal can be characterized by the graph Laplacian quadratic form

(39) $$\begin{array}{c} S\left(\hat{p}\right)={\hat{p}}^{T}L\hat{p}. \end{array}$$

It can be written as

(40) $$\begin{array}{c} S\left(\hat{p}\right)=\sum_{\left(i,j\in \varepsilon \right)}W_{ij}{\left({\hat{p}}_{j}-{\hat{p}}_{i}\right)}^{2}, \end{array}$$

where ε is the set of edges. The smaller the function value $S(\hat{p})$ is, the smoother the SNR difference of the cluster, especially when neighboring CUAVs connected by an edge with a large weight have similar values [15]. We can then represent the optimization problem as

(41) $$\begin{array}{c} \min_{\hat{p}}\;\frac{1}{2}{\|p_{0}-\hat{p}\|}_{2}^{2}+\frac{\rho }{2}{\hat{p}}^{T}L\hat{p}. \end{array}$$

To get the best estimated SNRs, we take the derivation of the formula, and get the optimal solution as

(42) $$\begin{array}{c} \hat{p}={\left(1+\rho L\right)}^{-1}p_{0}. \end{array}$$

**Algorithm 1** Space smoothing-based M2M4 SNR estimation algorithm1: **Input:** The received signal sample of each CUAV $\left\{y_{i1},y_{i2},\ldots ,y_{in}\right\}$.2: **Output:** The SNR of all the *N* CUAVs $\hat{p}=\left\{p_{1},p_{2},\ldots ,p_{N}\right\}$.
**Step 1: M2M4 SNR Estimation**
3: Calculate SNR of all the CUAVs with the traditional M2M4 algorithm in Section 6.1,  obtain $\left\{p_{01},p_{02},\ldots ,p_{0N}\right\}$.
**Step 2: Space Smoothing**
4: Adopt the Max-Min distance clustering algorithm, obtain the clustering result.5: With the clustering result, calculate the SNR of CUAVs in each cluster by Equation (34), then obtain the final estimated SNR $\hat{p}=\left\{{\hat{p}}_{1},{\hat{p}}_{2},\ldots ,{\hat{p}}_{N}\right\}$.

### 6.3. CHMM-Based Spectrum Sensing with SS-M2M4 SNR Estimation

The estimated SNRs can provide better SNR information for the CHMM-based spectrum sensing procedure, and thus we can obtain more accurate fusion weight in a heterogenous two-stage-fusion stage. Therefore, the CHMM-based spectrum sensing scheme with estimated SNRs achieves a higher detection probability, and the utilization of spectrum sensing can be further enhanced.

The proposed continuous hidden Markov model based spectrum sensing with space smoothing M2M4 SNR estimator can achieve good detection performance in CUAVNs, since it has some advantages over the existing HMM-based spectrum sensing method and the existing SNR estimators. The proposed method can either achieve better performance or be more suitable for UAV applications. The characteristics of these existed methods compared to the proposed method are summarized in Table 2.

## 7. Evaluation and Numerical Results

In this section, the validity of the approximation of folded normal distribution is firstly presented. Then, the detection performance of the proposed CHMM-based spectrum sensing scheme is evaluated by comparing with the non-CHMM ones. Next, we verify the effectiveness of the proposed SS-M2M4 estimator and further demonstrate the performance of the unified CHMM-based spectrum sensing with the SS-M2M4 estimator. Finally, we further consider mutipath effects in CUAVNs, and verify the effectiveness of the proposed scheme under the Rice channel.

### 7.1. Approximation of Folded Normal Distribution

The cluster heads adopt cyclostationary detection to sense the primary spectrum. Under the hypotheses $H_{1}$, the cyclostationary detection statistic *x* follows the folded normal distribution, which can be represented as

(43) $$\begin{array}{cc} f(x) & =\frac{1}{\sqrt{2\pi \frac{1}{M}}}\exp\left\{\frac{{\left(x-\sqrt{2\gamma }\right)}^{2}}{2\frac{1}{M}}\right\}+\frac{1}{\sqrt{2\pi \frac{1}{M}}}\exp\left\{\frac{{\left(x+\sqrt{2\gamma }\right)}^{2}}{2\frac{1}{M}}\right\},x>0. \end{array}$$

**Figure 5:**
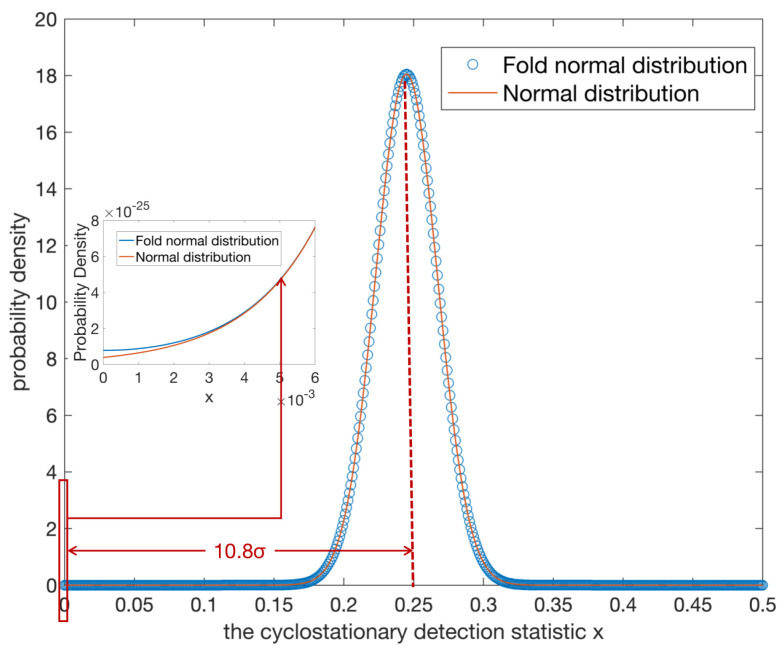
Approximation of folded distribution.

Figure 5 shows the probability density function of the cyclostationary feature under hypotheses $H_{1}$ when SNR=−15dB, M=2048. As shown in Figure 5, the folded normal distribution mainly coincides with the normal distribution. When *x* is less than 0.0025, which is already 10.8σ away from the mean, the two distributions start diverging. In fact, the two terms of folded normal distribution f(x) can be seen as two normal distributions with opposite means and the same variance. In spectrum sensing, when the channel is occupied, the mean $\sqrt{2\gamma }$ is away from 0, and it is much larger than the variance. Thus, when x>0, the second term of f(x) contributes little to the folded normal distribution. Therefore, in CUAVNs, folded normal distribution can be seen as its first term, that is, a normal distribution in the positive axis.

### 7.2. Performance of CHMM-Based Spectrum Sensing with Perfect SNR Estimation

In the simulations, the number of secondary CUAVs is set to 20. The CUAVs move according to the random walk mobility model [34], in which the maximum velocity of the CUAVs is 36km/h, and the sensing time is 20 μs. The range of the SNR in our CUAVNs is set as [−15, −3] dB according to [32,33,41]. Locations of secondary nodes lead to different SNRs, we assume that the maximum distance from the transmitter to the secondary UAV is about 1.5 to 2 times the minimum [1]. According to the large-scale fading calculation formula [40], assuming that the fading coefficient is 2, we can obtain 5 dB as a SNR range in our simulation, in other words, the maximum SNR difference of secondary users is within the range of 5 dB. We assume that all secondary CUAVs experience additive white Gaussian noise.

According to [42], the spectrum utilization rate below 3G (The Federal Communications Commission (FCC) of the United States and the European Union (EU) have set 2.4 GHz and 5.8 GHz as the band of civil UAVs. The EU also allocates 433 MHz and 863–870 MHz to UAVs. Similarly, China has set 840.5–845 MHz, 1438–1444 MHz and 2408–2440 MHz as that for UAVs. Compared to 5.8 GHz, more UAVs work on lower bands, below 3 GHz, thus the dilemma of spectrum scarcity is more serious on the below-3G bands) in Berkeley is about 0.3, therefore, we set the initial distribution of the spectrum state distribution as $\pi ={\left(0.7,0.3\right)}^{T}$, that is to say, the probability of spectrum presence is 0.3, and the probability of spectrum absence is 0.7. We assume a 1st order Markov chain, and the spectrum state at time *t* is known, where the distribution is either ${\left(1,0\right)}^{T}$ or ${\left(0,1\right)}^{T}$. As stated before, the spectrum utilization is around 0.3, the transition probability of absence to presence is thus set as 0.25, and in the same way, the transition probability of presence to presence is set as 0.35. Therefore, the transition matrix is A=[0.75,0.25;0.65,0.35]. Next, we use MATLAB to generate a spectrum state sequence with a length of 8000 under the parameters above. According to each single spectrum state (hidden state) and different clustering results, the simulated energy detection statistics $T_{E_{ij}}$ and cyclostationary statistics $T_{C,i}$ are obtained, respectively. Then corresponding observation values $o_{t}$ are calculated according to the two-step-fusion method.

**Figure 6:**
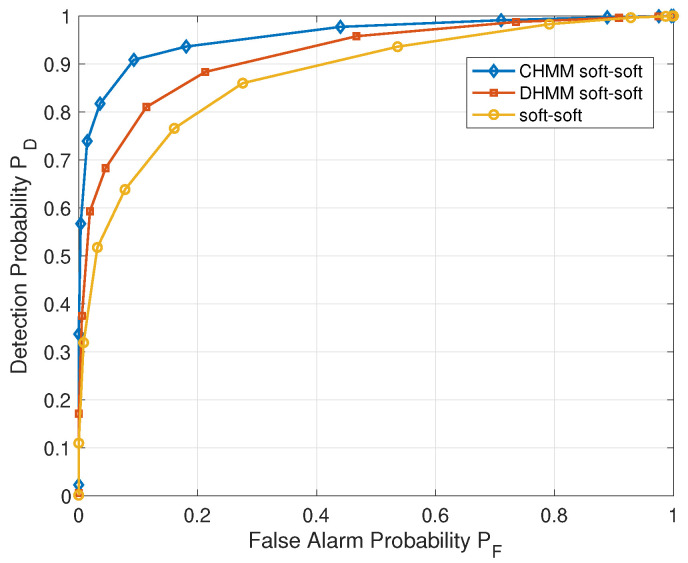
ROC of CHMM soft–soft scheme (the proposed CHMM-based heterogeneous two-stage fusion sensing scheme), DHMM soft–soft scheme and non-CHMM soft–soft scheme.

**Figure 7:**
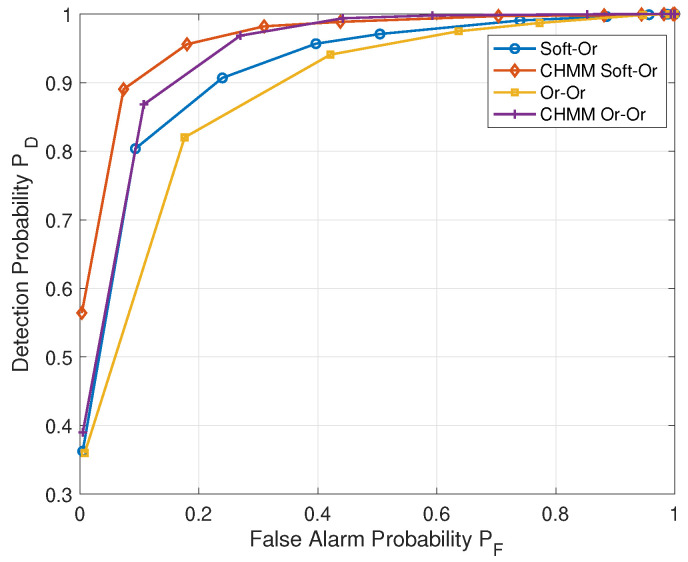
ROC of soft–or, or–or scheme and CHMM soft–or, or–or scheme.

Figure 6 and Figure 7 show the received operating curve (ROC) of the CHMM-based sensing method and non-CHMM-based ones under the AWGN channel with 20 CUAVs. In Figure 6, the soft–soft represents the heterogeneous two-stage sensing scheme represented in Section 2.2, in which both the intra-cluster fusion stage and the inter-cluster fusion stage adopt a soft combining rule [43]. It can be observed from Figure 6 that the soft–soft heterogeneous sensing scheme with CHMM predication outperforms the non-predication one (soft–soft). Due to CHMM avoiding the distortion caused by the discretization of DHMM, the proposed CHMM-based sensing scheme can further improve the detection probability compared with the DHMM-based soft–soft scheme, which adopts DHMM to model the sensing procedure. What stands out in Figure 6 is the achieved high detection probability of 0.91 when false alarm probability is around 0.1. A higher $P_{D}$ with small $P_{F}$ indicates that the proposed algorithm can offer the secondary CUAVs more opportunities to access the spectrum and maintain tolerable interruption to the primary user.

In addition, we also simulate the soft–or and or–or schemes [36] to verify the universality of the CHMM model for spectrum sensing in Figure 7, where soft–or means a soft combining rule at the intra-cluster fusion stage and/or a combining rule [43] at the inter-cluster fusion stage. Similarly, or–or means an or combining rule at both the intra-cluster fusion stage and the inter-cluster fusion stage. As shown in Figure 7, in addition to the good detection performance offered by the heterogeneous soft–soft scheme, the proposed CHMM can also achieve obvious improvement when it is implemented into the other two fusion schemes: or–or and soft–or. Then, with the help of a better sensing performance, the spectrum efficiency and throughput can be further improved.

### 7.3. Performance of CHMM-Based Spectrum Sensing with SS-M2M4 SNR Estimator

**Figure 8:**
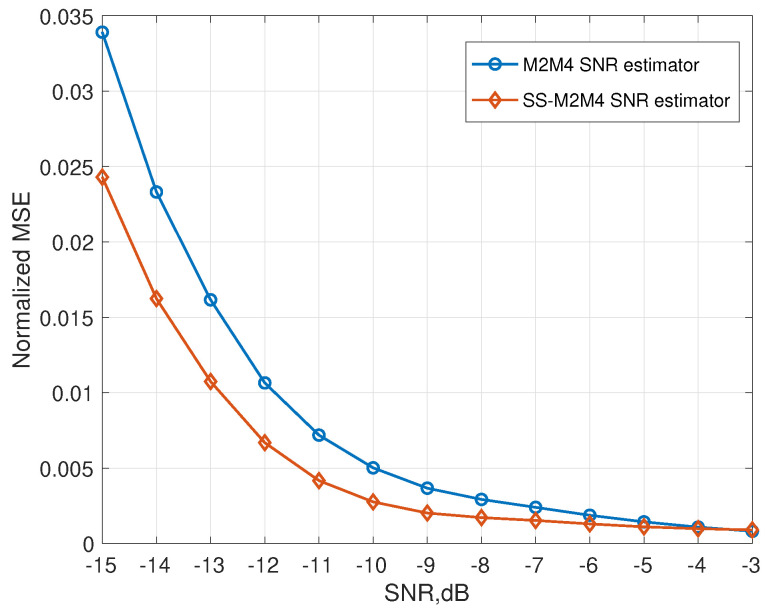
MSE of SS-M2M4 estimator and M2M4 estimator.

In this section, the simulation evaluation of our proposed SS-M2M4 in comparison with the existing M2M4 algorithm is presented. The simulation results with SNR in [−15, −3] are shown in Figure 8. Note that the SNR = −15 dB represents the SNR range from [−15, −10]. Similarly, −3 dB represents the range [−3, 2]. MSE is used to measure the effect of SNR estimation, and the calculation scheme of MSE is given as

(44) $$\begin{array}{c} MSE=\frac{1}{N}\sum_{i=1}^{N}{\left(p_{i}-{\hat{p}}_{i}\right)}^{2}, \end{array}$$

where ${\hat{p}}_{i}$ represents the estimated SNR of the *i*th CUAV, $p_{i}$ represents the actual SNR of the *i*th CUAV. Figure 8 shows that the proposed scheme offers a good SNR error decrease. It can be seen that Figure 8 also shows that the proposed scheme can improve the performance of SNR estimation up to 3dB (when a $2.3\times 10^{-3}$MSE is required, the M2M4 estimator needs the actual SNR to be −6 dB, but SS-M2M4 only needs it to be −9 dB). An accurate SNR estimator can further help the CUAV get a better detection performance. Consequently, it is reasonable to expect good detection performance when the CHMM-based spectrum sensing adopts the novel SNR estimation algorithm.

**Figure 9:**
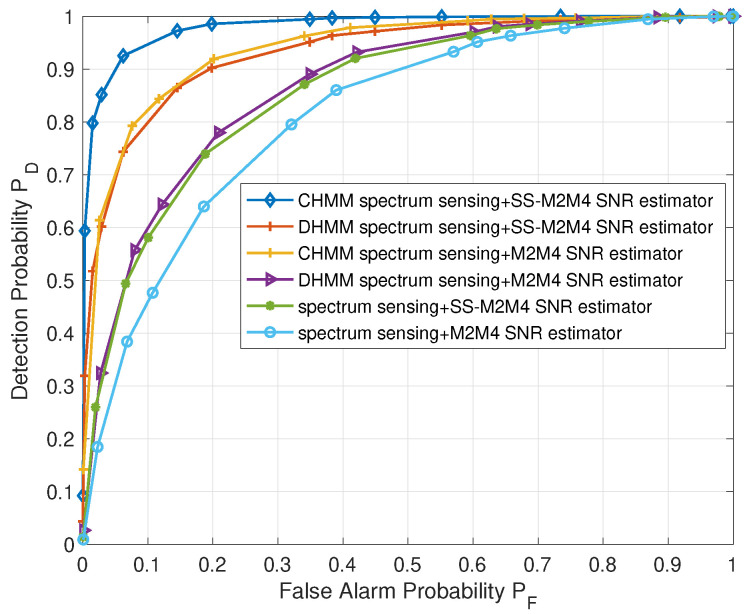
Detection probability versus false alarm probability.

Figure 9 shows the CHMM-based spectrum sensing with the SS-M2M4 estimator significantly outperforming the original ones, which either do not use the CHMM model (spectrum sensing + SS-M2M4 SNR estimator) or employ the M2M4 estimator (CHMM spectrum sensing + M2M4 SNR estimator), or neither (spectrum sensing + M2M4 SNR estimator). Specifically, when the false alarm is 0.1, the unified scheme achieves a detection probability of 0.95, while the CHMM-based spectrum sensing with the original SNR estimator (CHMM spectrum sensing + M2M4 SNR estimator) can only achieve around 0.82. Besides, the unified scheme can further enhance the detection performance compared with the DHMM-based spectrum sensing with the proposed SS-M2M4 (DHMM spectrum sensing + SS-M2M4 SNR estimator), and the DHMM-based spectrum sensing with the proposed SNR estimator can also outperform the DHMM-based spectrum sensing with the original SNR estimator (DHMM spectrum sensing + M2M4 SNR estimator). In other words, the proposed SNR estimator has a universality to a different spectrum sensing method and the unified scheme can utilize the spectrum more efficiently and maintain a tiny interference to the primary user.

### 7.4. Performance of CHMM-Based Spectrum Sensing with SS-M2M4 SNR Estimator under Rice Channel

**Figure 10:**
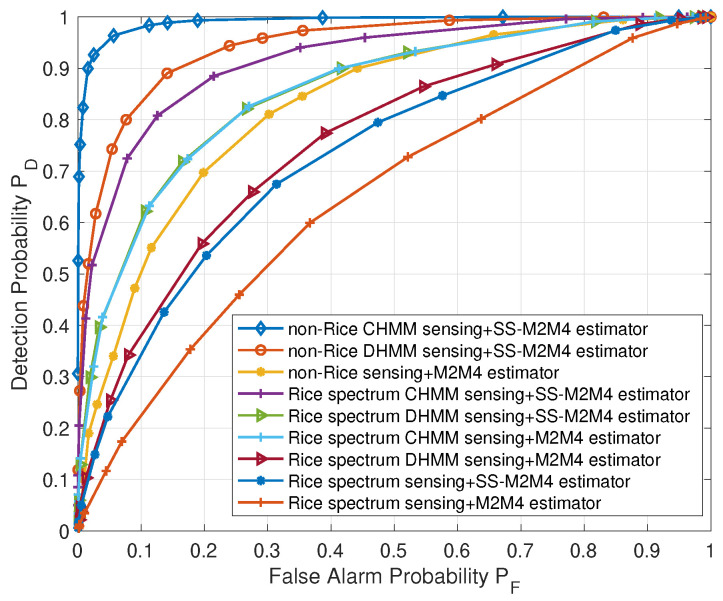
Detection probability versus false alarm probability under the Rice channel.

In the above simulation, we assume the AWGN channel and verify the effectiveness of the proposed scheme under the AWGN channel. In this section, we have further considered the Rice channel when mutipath effects have been introduced in UAV applications [44]. The simulation of the proposed scheme under the Rice channel is shown in Figure 10, according to [44], we set the Rician factor as K = 10. Figure 10 shows that even under the Rice channel, the proposed method (Rice CHMM sensing+SS-M2M4 estimator) can obtain better performance compared with DHMM-based spectrum sensing with the proposed SS-M2M4 SNR estimator (Rice DHMM sensing + SS-M2M4 estimator), CHMM-based spectrum sensing with M2M4 SNR estimator (Rice CHMM sensing+M2M4 estimator), and other methods. Although the detection probability under the Rice channel is lower than that under the AWGN channel, the proposed scheme can also achieve better performance than other schemes under the Rice channel, in other words, our scheme is effective in CUANs.

## 8. Conclusions

In this paper, we consider modeling primary user states as a Markov chain, and propose a spectrum sensing scheme based on a continuous hidden Markov model with perfect SNR estimation. We derive the detection probability and false alarm probability of the heterogenous-fusion clustered CUAVNs. Taking the similarity of the SNRs of CUAVs in the same cluster into account, we propose a space smoothing based SNR estimator for sensing in CUAVNs to offer a more accurate SNR to the proposed sensing method. Simulation results show that the unified CHMM-based sensing scheme with the proposed SNR estimator enhances the sensing performance considerably.

The work can be further extended in the following aspects in our future research. First, in the proposed CHMM based spectrum sensing method, the hidden spectrum chain is modeled by first-order Markov chain, and it has not made full use of the existing historical information. In the future research, the high-order Markov chain can be used to model the spectrum sensing process to further improve the accuracy of prediction. Second, in this paper, we considered cognitive UAV networks with a single primary user. Recently, cognitive UAV networks with multi primary users [33] have been proposed to enhance the spectrum utilization. Thus, we can explore our work in multi-PUs CUAVNs in the future.

## Figures and Tables

**Table 1:** Notations.

Notation	Description	Notatiom	Description
*a*	the amplitude of the transmitted signal	$T_{i}$	the likelihood ratio test statistic of the *i*th cluster
$f_{c}$	the carrier frequency of the transmitted signal	$T_{ij}$	the cyclostationary detection statistics or energy detection statistics
φ	the carrier phase offset of the transmitted signal	$\omega_{ij}$	weight of $T_{E_{ij}}$
*M*	the number of sampling point	$\rho_{i}$	weight of $T_{C_{i}}$
α	the cyclic frequency of the received signal	$\gamma_{C_{i}}$	the SNR of the *i*th cluster head
$T_{C}$	the test statistic for the first-order cyclostationary detection	$\gamma_{E_{ij}}$	the SNR of the *j*th node in the *i*th cluster
$T_{E}$	the test statistic for energy detector	*L*	the Laplacian matrix of the cluster heads topology
$T_{ic}$	the statistics of the *i*th cluster	$\alpha_{l}$	step size
$T_{C_{i}}$	the cyclostationary detection statistics of the *i*th cluster head	*d*	maximum node degree of the cluster graph
$T_{E_{ij}}$	the energy detection statistics of the *j*th the node in the *i*th cluster	$\delta_{h_{i}}$	the weight of the *i*th cluster head
*K*	the number of CUAVs in the *i*th cluster	$T^{*}$	the final consensus
$K_{E}$	the number of cluster members in the *i*th cluster	*W*	the consensus weight matrix

**Table 2:** The characteristics of the existing methods.

Methods	Characteristics	Examples
DHMM-based spectrum sensing	Errors caused by quantization degrade detection performance	Suguna et al. [21], Eltom et al. [22]
CHMM-based spectrum sensing	Centralized spectrum sensing and lack of dynamicity, not suitable for CUAVNs	Halaseh et al. [23], Cheng et al. [24]
Deep learning-based spectrum sensing	Poor interpretability and missing of the sensing term caused by the large delay	Xie et al. [16], Xie et al. [17]
SNR estimator with pre-knowledge	Hard to get pre-knowledge of channel in UAVs applications	Raza et al. [26], Manesh et al. [27]
SNR estimator designed for specific signals	Not generalized for UAVs applications	He et al. [28], Chen et al. [29]
Deep learning-based SNR estimator	Missing of the sensing term caused by the long estimation time	Ngo et al. [31], Yang et al. [32]

## Data Availability

Not applicable.

## Appendix A. Formulas of the Updated CHMM Parameters

The parameters of the CHMM [39] λ=(π,A,μ,Σ,C) can be updated with parameters $\gamma_{t}\left(i\right)$, $\xi_{t}\left(i,j\right)$ and $\gamma_{t}\left(j,m\right)$ as follows

(A1) $$\begin{array}{c} \pi_{i}=\gamma_{t}\left(i\right), \end{array}$$

(A2) $$\begin{array}{c} a_{ij}=\frac{\sum_{t=1}^{T-1}\delta_{t}\left(i,j\right)}{\sum_{t=1}^{T-1}\gamma_{t}\left(i\right)}, \end{array}$$

(A3) $$\begin{array}{c} c_{j,m}=\frac{\sum_{t=1}^{T}\gamma_{t}\left(j,m\right)}{\sum_{t=1}^{T}\sum_{n=1}^{2}\gamma_{t}\left(j,n\right)}, \end{array}$$

(A4) $$\begin{array}{c} \mu_{j,m}=\frac{\sum_{t=1}^{T}\gamma_{t}\left(j,m\right)o_{t}}{\sum_{t=1}^{T}\gamma_{t}\left(j,m\right)}, \end{array}$$

(A5) $$\begin{array}{c} \Sigma_{j,m}=\frac{\sum_{t=1}^{T}\gamma_{t}\left(j,m\right)\left(o_{t}-\mu_{j,m}\right){\left(o_{t}-\mu_{j,m}\right)}^{T}}{\sum_{t=1}^{T}\gamma_{t}\left(j,m\right)}. \end{array}$$

## Appendix B. Proof of Distribution of the Fusion Value

In this section, we derive the distribution of the fusion result *T*, where $T=T_{E}+T_{C}$. To obtain the fusion result *T*, we first add the energy detection result $T_{Eij}$ and the cyclostationary detection result $T_{C_{i}}$, respectively, and then calculate the summation of the two different detection results. When *M* is large enough, the sum of the energy detection $T_{E}$ follows normal distribution written as

(A6) $$\begin{array}{c} f\left(T_{E}\right)=\frac{1}{\sqrt{2\pi }{\sigma_{E}}^{2}}e^{-\frac{{\left(T_{E}-\gamma \right)}^{2}}{2{\sigma_{E}}^{2}}}, \end{array}$$

where mean $\gamma =\sum_{i=1}^{n}\sum_{j=1}^{K_{E}}\delta_{h_{i}}/\left\{2\left(1+\gamma_{E_{ij}}\right)\right\}$, variance ${\sigma_{E}}^{2}=\sum_{i=1}^{n}\sum_{j=1}^{K_{E}}{\delta_{h_{i}}}^{2}/\left\{2M{\left(1+\gamma_{E_{ij}}\right)}^{2}\right\}$. To obtain the distribution which the sum of cyclostationary detection follows, we do the following derivation. For simplicity, x denotes one cyclostationary detection result $T_{C_{i}}$, y denotes another cyclostationary detection result $T_{C_{j}}$, and the distribution of *x* and *y* can be represent as

(A7) $$\begin{array}{c} f\left(x\right)=\frac{2}{\sqrt{2\pi }\sigma }e^{-\frac{x^{2}}{2\sigma^{2}}}, \end{array}$$

(A8) $$\begin{array}{c} f\left(y\right)=\frac{2}{\sqrt{2\pi }\sigma }e^{-\frac{y^{2}}{2\sigma^{2}}}, \end{array}$$

with the same mean 0 and the same variance $\sigma^{2}=\frac{2}{M}$, z=ax+by is defined as the sum of x and y, we can derive the distribution of *z* as

(A9) $$\begin{array}{cc} f(z) & =\int_{0}^{+\infty }\frac{1}{a}f_{x}\left(\frac{z-by}{a}\right)f_{y}\left(y\right)dy\\ \; & =\int_{0}^{+\infty }\frac{1}{a}\frac{2}{\sqrt{2\pi }\sigma }\exp\left\{-\frac{{\left(\frac{z-by}{a}\right)}^{2}}{2\sigma^{2}}\right\}\frac{2}{\sqrt{2\pi }\sigma }\exp\left\{-\frac{y^{2}}{2\sigma^{2}}\right\}dy\\ \; & ={\left(\frac{2}{\sqrt{2a\pi }\sigma }\right)}^{2}\exp\left\{-\frac{\frac{z^{2}}{a^{2}+b^{2}}}{2\sigma^{2}}\right\}\int_{0}^{+\infty }\exp\left\{-\frac{{\left(\sqrt{a^{2}+b^{2}}y-\frac{bz}{\sqrt{a^{2}+b^{2}}}\right)}^{2}}{2a^{2}\sigma^{2}}\right\}dy\\ \; & ={\left(\frac{2}{\sqrt{2a\pi }\sigma }\right)}^{2}\int_{-\frac{bz}{\sqrt{2}a\sqrt{a^{2}+b^{2}}\sigma }}^{+\infty }\exp\left\{-t^{2}\right\}dt\frac{\sqrt{2}a\sigma }{\sqrt{a^{2}+b^{2}}}\exp\left\{-\frac{z^{2}}{2\left(a^{2}+b^{2}\right)\sigma^{2}}\right\}\\ \; & ={\left(\frac{2}{\sqrt{2a\pi }\sigma }\right)}^{2}\frac{\sqrt{2}a\sigma }{\sqrt{a^{2}+b^{2}}}\exp\left\{-\frac{z^{2}}{2\left(a^{2}+b^{2}\right)\sigma^{2}}\right\}\int_{-\infty }^{+\infty }\exp\left\{-t^{2}\right\}dt\\ \; & =\frac{4}{\sqrt{2\pi \left(a^{2}+b^{2}\right)\sigma^{2}}}\exp\left\{-\frac{z^{2}}{2\left(a^{2}+b^{2}\right)\sigma^{2}}\right\}, \end{array}$$

where $t=\frac{\sqrt{a^{2}+b^{2}}y-\frac{bz}{\sqrt{a^{2}+b^{2}}}}{\sqrt{2}a\sigma },y=\frac{\sqrt{2}a\sigma t+\frac{bz}{\sqrt{a^{2}+b^{2}}}}{\sqrt{a^{2}+b^{2}}}$. From the above proof, we can conclude that the distribution of *z* is similar with *x* and *y*, but there exist some differences: the coefficient has doubled, the variance is the sum of $\sigma_{x}$ and $\sigma_{y}$. Thus $T_{C}$ follows the following distribution

(A10) $$\begin{array}{c} f\left(T_{C}\right)=\frac{2n}{\sqrt{2\pi {\sigma_{C}}^{2}}}\exp\left\{-\frac{{T_{C}}^{2}}{2{\sigma_{C}}^{2}}\right\}. \end{array}$$

After obtaining the distribution of cyclostationary detection, we can further derive the distribution of the fusion result *T* as follows,

$$\begin{array}{cc} f(T) & =\int_{0}^{+\infty }f_{T_{E}}\left(T-T_{C}\right)f_{T_{C}}\left(T_{C}\right)dT_{C}\\ \; & =\frac{2n}{\sqrt{2\pi {\sigma_{E}}^{2}}\sqrt{2\pi {\sigma_{C}}^{2}}}\int_{0}^{+\infty }\exp\left\{-\frac{{T_{C}}^{2}}{2\sigma_{C}^{2}}\right\}\exp\left\{-\frac{{\left(T-T_{C}-\gamma \right)}^{2}}{2{\sigma_{E}}^{2}}\right\}dT_{C}\\ \; & =\frac{2n}{2\pi \sqrt{{\sigma_{E}}^{2}\sigma_{C}^{2}}}\exp\left\{-\frac{{\left(T-\gamma \right)}^{2}}{2\sigma^{2}}\right\}\int_{0}^{+\infty }\exp\left\{\frac{-{\left[\sigma T_{C}-\frac{{\sigma_{C}}^{2}\left(T-\gamma \right)}{\sigma }\right]}^{2}}{2{\sigma_{E}}^{2}{\sigma_{C}}^{2}}\right\}dT_{C} \end{array}$$

(A11) $$\begin{array}{cc} \; & =\frac{2n}{2\pi \sqrt{{\sigma_{E}}^{2}\sigma_{C}^{2}}}\exp\left\{-\frac{{\left(T-\gamma \right)}^{2}}{2\sigma^{2}}\right\}\frac{2\sqrt{{\sigma_{E}}^{2}{\sigma_{C}}^{2}}}{\sigma }\int_{0}^{+\infty }\exp\left\{-t^{2}\right\}dt\\ \; & =\frac{n}{\sqrt{2\pi \sigma^{2}}}\exp\left\{-\frac{{\left(T-\gamma \right)}^{2}}{2\sigma^{2}}\right\}erfc\left(\frac{\sigma_{C}\left(T-\gamma \right)}{\sqrt{2{\sigma_{E}}^{2}\sigma^{2}}}\right), \end{array}$$

where *n* is number of the clusters, $t=\left[\sqrt{{\sigma_{E}}^{2}+{\sigma_{C}}^{2}}T_{C}-\frac{{\sigma_{C}}^{2}\left(T-\gamma \right)}{\sqrt{{\sigma_{E}}^{2}+{\sigma_{C}}^{2}}}\right]/\sqrt{2{\sigma_{E}}^{2}{\sigma_{C}}^{2}}$, ${\sigma_{C}}^{2}=\sum_{i=1}^{n}2M{\delta_{h_{i}}}^{2}\gamma_{C_{i}}$, $\sigma^{2}={\sigma_{E}}^{2}+{\sigma_{C}}^{2}$. Finally, the distribution of the fusion result *T* under hypotheses $H_{0}$ can be obtained as

(A12) $$\begin{array}{c} f\left(T\right)=\frac{n}{\sqrt{2\pi \sigma^{2}}}\exp\left\{-\frac{{\left(T-\gamma \right)}^{2}}{2\sigma^{2}}\right\}erfc\left(\frac{\sigma_{C}\left(T-\gamma \right)}{\sqrt{2{\sigma_{E}}^{2}\sigma^{2}}}\right). \end{array}$$
